# Genetic diversity of *Mycobacterium tuberculosis* isolated from tuberculosis patients in the Serengeti ecosystem in Tanzania

**DOI:** 10.1016/j.tube.2014.11.006

**Published:** 2015-03

**Authors:** Erasto V. Mbugi, Bugwesa Z. Katale, Keith K. Siame, Julius D. Keyyu, Sharon L. Kendall, Hazel M. Dockrell, Elizabeth M. Streicher, Anita L. Michel, Mark M. Rweyemamu, Robin M. Warren, Mecky I. Matee, Paul D. van Helden

**Affiliations:** a Department of Biochemistry, Muhimbili University of Health and Allied Sciences, P. O. Box 65001 Dar es Salaam, Tanzania; b Departments of Microbiology and Immunology, Muhimbili University of Health and Allied Sciences, P.O. Box 65001 Dar es Salaam, Tanzania; c DST/NRF Centre of Excellence for Biomedical Tuberculosis Research/ Medical Research Council (MRC) Centre for Tuberculosis Research, Division of Molecular Biology and Human Genetics, Faculty of Health Sciences, Stellenbosch University, P. O. Box 19063, Tygerberg, 7505, South Africa; d Tanzania Wildlife Research Institute (TAWIRI), P.O. Box 661, Arusha, Tanzania; e The Royal Veterinary College, Royal College Street, London, NW1 0TU, United Kingdom; f Department of Immunology and Infection, London School of Hygiene and Tropical Medicine, Keppel Street, London WC1E 7HT, United Kingdom; g Department of Veterinary Tropical Diseases, Faculty of Veterinary Science, University of Pretoria, South Africa; h Southern African Centre for Infectious Disease Surveillance, Sokoine University of Agriculture, Morogoro, Tanzania

**Keywords:** *Mycobacterium tuberculosis*, Genotyping, Human–animal interface, Serengeti ecosystem

## Abstract

This study was part of a larger cross-sectional survey that was evaluating tuberculosis (TB) infection in humans, livestock and wildlife in the Serengeti ecosystem in Tanzania. The study aimed at evaluating the genetic diversity of *Mycobacterium tuberculosis* isolates from TB patients attending health facilities in the Serengeti ecosystem. DNA was extracted from 214 sputum cultures obtained from consecutively enrolled newly diagnosed untreated TB patients aged ≥18 years. Spacer oligonucleotide typing (spoligotyping) and Mycobacterium Interspersed Repetitive Units and Variable Number Tandem Repeat (MIRU-VNTR) were used to genotype *M. tuberculosis* to establish the circulating lineages. Of the214 *M. tuberculosis* isolates genotyped, 55 (25.7%) belonged to the Central Asian (CAS) family, 52 (24.3%) were T family (an ill-defined family), 38 (17.8%) belonged to the Latin American Mediterranean (LAM) family, 25 (11.7%) to the East-African Indian (EAI) family, 25 (11.7%) comprised of different unassigned (‘Serengeti’) strain families, while 8 (3.7%) belonged to the Beijing family. A minority group that included Haarlem, X, U and S altogether accounted for 11 (5.2%) of all genotypes. MIRU-VNTR typing produced diverse patterns within and between families indicative of unlinked transmission chains. We conclude that, in the Serengeti ecosystem only a few successful families predominate namely CAS, T, LAM and EAI families. Other types found in lower prevalence are Beijing, Haarlem, X, S and MANU. The Haarlem, EAI_Somalia, LAM3 and S/convergent and X2 subfamilies found in this study were not reported in previous studies in Tanzania.

## 1 Background

Tuberculosis (TB) caused by *Mycobacterium tuberculosis* (*Mtb*) is the second major cause of death from infectious diseases worldwide [1]. Over the last two decades, molecular typing methods such as IS*6110*-RFLP [2], spoligotyping [3] and MIRU-VNTR [4] have been applied and have revolutionised our understanding of the epidemiology of TB, by providing novel insights into the genetic diversity and population structure of *M. tuberculosis* complex (MTBC) [5]. Epidemiological data generated through genotyping has been used extensively to further the understanding of TB disease dynamics [6]. For example, at the individual level, cases of recurrence or treatment failure can be explained in terms of reactivation of the same strain, exogenous re-infection or due to polyclonal infection [7]. At a population level, the origins and transmission dynamics of outbreaks can be determined [8–10]; while at global level, TB genotypic lineages have been defined and used to monitor their geographical distribution and spread [6]. A crucial aspect of any TB control program is the ability to quantify the contribution of transmission in order to inform policy makers to direct resources to identify infectious cases to prevent further spread of infection as well as to implement preventative therapy for those who have been infected (children and HIV positive individuals). The CAS family of *M. tuberculosis* strains are dominant in Tanzania [11–13] with little variations over time period [14,15] with some anti-tuberculosis drug resistance and multidrug resistance [15]. Although these previous studies have been carried in northern Tanzania (4) and Dar es Salaam (1) none has targeted the Serengeti ecosystem and the earlier studies focused largely on TB and its association with HIV/AIDS. However, one cannot conclude that every location in Tanzania is represented by this earlier data which therefore provides a justification for studies in new locations, where findings might potentially provide data that could influence TB control strategies in the country.

In this study we used molecular epidemiological tools to describe the genetic diversity of mycobacteria in the Serengeti ecosystem where humans, livestock and wildlife are in close contact with the possibility of cross-transmission [16]. Specifically, the genotyping was achieved using spoligotyping and MIRU-VNTR typing methods. We report on the genetic diversity of *M*. *tuberculosis* isolated from tuberculosis patients resident in three sub-districts of the Serengeti ecosystem.

## 2 Materials and methods

### 2.1 Study design and settings

**Figure 1:**
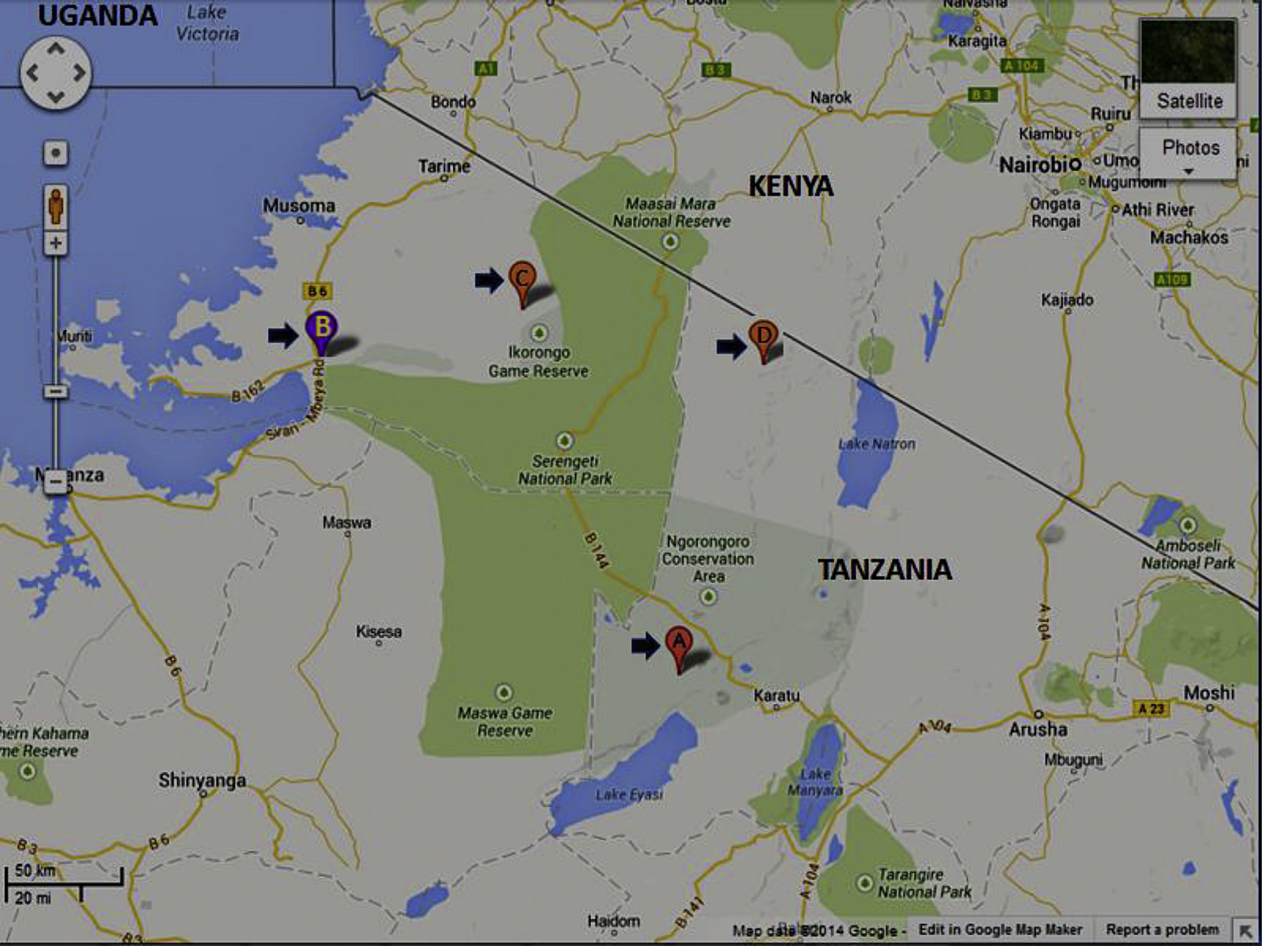
Map of the Serengeti ecosystem comprising of the Serengeti National Park, Ngorongoro Conservation Area, Ikorongo-Grumeti Game Reserves, Maswa Game Reserve. The District Designated Hospitals and Health Centres are indicated by arrows and marked by letters A: Endulen Heath Centre; B: Bunda DDH; C: Mugumu DDH; D: Waso DDH.

This cross-sectional study was conducted in focal health facilities serving three districts of Bunda, Ngorongoro and Mugumu-Serengeti in the Serengeti ecosystem where TB screening is done regularly (Figure 1). The population densities for the three districts according to Tanzanian population statistics (2013) are Bunda (108.3 persons/km²), Serengeti (22.4 persons/km²) and Ngorongoro (11.2 persons/km²) with unpublished and limited information on the incidences of TB and HIV in these areas. These centres included District Designated Hospitals (DDH) in Bunda, Serengeti (Mugumu), Ngorongoro (Waso) and Endulen (in the Ngorongoro Conservation Area). The Bunda District Designated Hospital (DDH) provides health services to nearby villages of neighbouring districts, such as Magu district (Lamadi village very close to Bunda) of Mwanza alongside the Serengeti National Park, some villages of Musoma district and other nearby districts of the Mara region. All patients with symptoms suggestive of TB presenting at these facilities during the study period (October 2010–November 2012) were eligible for the study. Only patients who gave written informed and signed consent forms were enrolled in the study.

### 2.2 Sample collection

Sputum samples from self-reporting TB suspects were consecutively collected in transport medium, cetyl-pyridinium chloride (CPC) [17] from October 2010 to November 2012. A total of 472 sputum samples were collected from individuals presenting with TB symptoms. The sputum smears were Ziehl Neelsen-stained and examined microscopically for acid-fast bacilli (AFB) at the health centres. Two hundred and thirty-seven (237, 50.2%) were AFB smear-positive. All sputum samples were then transported in cetyl-pyridinium chloride (CPC) to the Central TB Reference Laboratory (CTRL) in Dar es Salaam for mycobacterial culture within 7 days of collection.

### 2.3 Sputum sample processing

During sample processing, the sputum-CPC mixture was concentrated by centrifuging at 4000 rpm for 15 min. The supernatants were poured off into a splash proof container. Twenty millilitres (20 ml) of sterile distilled water was added to the sediments and the pellets were suspended by inverting the tubes several times, and then centrifuged at 3500 rpm for 15 min. The supernatant was removed; the pellets were used for culture. On culture 214 (out of 237 sputa, 90.3%) yielded *M. tuberculosis* colony growths which were available for DNA extraction and subsequent molecular analysis.

### 2.4 Culture and identification

Two Löwenstein-Jensen slants, one containing 0.75% glycerol and the other 0.6% pyruvate were inoculated with the sediments and incubated at 37 °C and growth examined weekly for 8 weeks whereby cultures with no growth after eight weeks were considered negative.

#### 2.4.1 DNA extraction procedures

Extraction of mycobacterial DNA was performed by boiling a loop full of bacteria in 100 μL H_2_O at 80 °C for 60 min. Crude DNA extracts were stored at −20 °C until when spoligotyping and MIRU-VNTR typing were performed.

### 2.5 Spoligotyping

A commercially available spoligotyping kit (Isogen, Bioscience BV, Maarssen, The Netherlands) was used for spoligotyping as previously described by Kamerbeek et al. [3]. This PCR-based fingerprinting method detects the presence or absence of 43 variable spacer sequences situated between short direct repeat (DR) sequences in the *M. tuberculosis* genome. The DNA from reference *M. tuberculosis* H37Rv and *Mycobacterium bovis* BCG clones were used as positive controls while autoclaved ultrapure water was used as a negative control. Visualization of presence (black squares) or absence (blank squares) of variable spacer sequences on film was achieved after incubation with streptavidin-peroxidase and detection of hybridized DNA using enhanced chemiluminescent ECL (Amersham, Little Chalfont, United Kingdom) detection liquid followed by exposure to X-ray film (Hyperfilm ECL; Amersham) as per manufacturer's instructions (GE Healthcare Life Sciences). Resulting spoligotypes were reported in octal and binary formats (Table 3) and compared to existing patterns in an international spoligotyping database profiles (SpolDB4.0) [18] available at http://www.pasteur-guadeloupe.fr:8081/SITVITDemo/. Spoligotype patterns were grouped as spoligotype international types (SITs) if they shared identical spoligotype patterns with patterns present in the existing database. In previous studies, isolates which could not be assigned to specific SITs were referred to as orphans [19–21]; in our study we decided to name the isolates with no SITs assigned as ‘Serengeti strains’. Spoligotype families were assigned as previously described [18,22].

### 2.6 MIRU-VNTR typing

The standardized 24 loci MIRU-VNTR typing protocol by Supply et al. [23]was followed using primers that amplify 24 polymorphic loci on the mycobacterial genome per DNA isolate. All Beijing genotype isolates and a selection of isolates representing the spoligotype EAI5, CAS1_Kili, CAS1_DELHI and LAM11_ZWE families were genotyped using this method. In brief, 2 μl of mycobacterial DNA was added to a final volume of 25 μl containing 8.375 μl of free RNase water (Qiagen, USA), 5 μl of Q solution, 2.5 μl of 10*x* buffer, 2 μl of 1.5 mM MgCl_2_ (Roche, USA), 4 μl of 0.2 mM dNTPs (Promega, WI USA), 1 μl primer and 0.125 μl of HotStar Taq polymerase (1U). The PCR conditions included three stages: initial denaturation at 95 °C for 15 min (Stage 1), second denaturation at 94 °C for 1 min, annealing at 62 °C for 1 min, initial extension for 1 min at 72 °C(Stage 2) and final extension at 72 °C for 10 min followed by cooling to 4 °C prior to analysis (Stage 3). A 45-cycles PCR was done on Veriti™ 96-well Thermal Cycler (Applied Bio system, Singapore). The laboratory *M. tuberculosis* H37Rv reference strain DNA was used as positive control and DNA-free water as a negative control. Amplification products were electrophoretically fractionated in 1% agarose gel (SeaKem^®^ LE) in 1*x* SPE buffer at 160 V for 4 h to allow maximum fragment size separation for clear discrimination. The number of tandem repeat units present at each locus was calculated from the size of DNA fragments according to a standardized table (http://www.MIRU-VNTRplus.org). The results were expressed in digital format where each number represented the number of repeat copies at a particular locus. Phylogenetic analysis and creation of dendograms was done using MIRU-VNTR*plus* (http://www.MIRU-VNTRPlus.org/) to generate a categorical based NJ-Tree dendrogram to enable comparison of strain genotypes within the study area [24,25] in an attempt to establish transmission links.

## 3 Results

### 3.1 Mycobacterial cultures

During the study period sputum samples were collected from 472 individuals presenting with TB symptoms. Of these, 237 (50.2%) were smear-positive and when cultured 214 grew *M. tuberculosis* on LJ media, with colonies that provided DNA which was available for genotyping.

### 3.2 Spoligotyping and distribution of spoligotypes by district

As shown in Table 1, 88.3% (189 out of 214 isolates) of the spoligotypes could be grouped into 9 known spoligotype families, while 25 (11.7%) were ‘Serengeti strains’. The Serengeti type strains resemble the CAS strain family, but have not previously been reported in SpolDB4. The Central Asian Strain family (CAS) accounted for 25.7% (*n* = 55) of all isolates followed by an ill-defined (T) family that accounted for 52 (24.3%) isolates. The Latin American Mediterranean (LAM) family accounted for 38 (17.8%) isolates while the East African-Indian family accounted for 25 (11.7%) isolates. Eight (3.7%) isolates belonged to the Beijing family. The rest of the families were minor and comprised of Haarlem (8, 2.8%), X (1, 0.5%), U (0.9%), S (1, 0.5%) and MANU (1, 0.5%). Breakdown of spoligotypes by districts (Table 1) indicated 33 (60%) of the CAS family overrepresented in Ngorongoro with Serengeti and Bunda districts accounting for 11 (20%) of the strain type each. A relatively high proportion (40.4%) of T family strains was found in Bunda district compared to Serengeti (32.7%) and Ngorongoro (26.9%). As regards the LAM family, nearly half (47.4%) of the strains in this family were found in Ngorongoro, followed by Bunda (36.8%) and Serengeti (15.8%). The rest of the strains found in small proportions were considered minor (Table 1). However, the high number for Bunda could be explained by the relatively larger sample size (almost double, that of Serengeti). The distribution of strain families by district is reflected in Table 1 and the finer categorization into subfamilies is presented in Table 2.

### 3.3 Distribution of strain subfamilies

Comparisons at district levels were more convenient to address spatial dominance of these subfamilies, considering the differences in sample sizes by districts. Assignment of spoligotype patterns to families revealed the CAS1_Kili subfamily to be predominant constituting 17.8% (38/214) of all spoligotype patterns (Table 2). Most of the CAS1_Kili strains were found in Ngorongoro (68.4%, 26/34) followed by Bunda (23.7%, 9/38) and Serengeti (7.9%, 3/38). The LAM11_ZWE strains were the second most dominant (13.6%, 29/214), with highest proportions in Bunda (41.4%) followed by Ngorongoro (37.9%) and then Serengeti (20.7%). These dominant subfamilies were followed by the T1 family (10.7%), EAI5 (9.3%), CAS1_DELHI (6.5%), T2 (5.6%), T3_ETH (5.1%), Beijing (3.7%) and LAM9 (3.3%). Other subfamilies were variably found in smaller proportions and included in this group were the EAI5 or EAI3 (1.9%), H3 (1.4%), T2-Uganda (1.4%), H1 (0.9%), CAS (0.9%), T3 (0.9%) and other strains (Table 2). Among the strains that found to be in small proportions, the Haarlem (2.8%), EAI_Somalia (0.5%), LAM3 and S/convergent (0.5%) and X2 (0.5%) subfamilies were not reported in previous studies in Tanzania. About 11.7% of our isolates were ‘Serengeti strains’. Representative isolates indicating strain family (or clades), SITs, their octal codes and the webdings format for absence or presence of specific spacers along the DR (direct repeat) region of the *M. tuberculosis* gene are shown in Table 3.

### 3.4 MIRU-VNTR typing and phylogenetics

**Figure 2:**
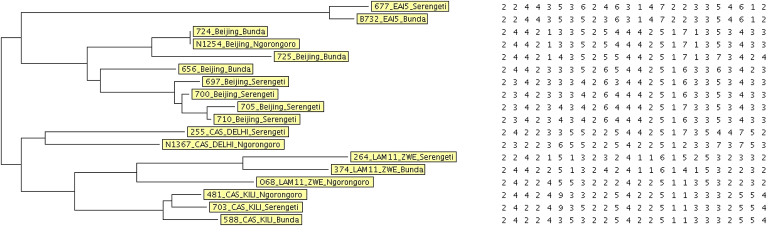
A NJ-Tree dendrogram of 18 representative samples from MIRU-VNTR typing showing relationships among families. The categorical based NJ-phylogenetic tree was generated from MIRU-VNTRPlus-24 (http://www.miru-vntrplus.org/). Selected strains were used from each constituent site in the ecosystem to assess whether there was a defined transmission link for the circulating strains in the ecosystem.

Phylogenetic relationship between subfamilies indicative of dynamics of transmission from standard 24-loci MIRU-VNTR typing of selected few isolates is presented in a dendrogram (Figure 2). The standard 24-loci MIRU-VNTR tying results were compared with their respective spoligotyping results (Table 4). The MIRU-VNTR typing patterns of polymorphisms at different loci along the mycobacterial genome are clearly reflected. While spoligotype patterns constituting the same family are largely identical, the MIRU-VNTR patterns often differed in at least one locus. Most related families and subfamilies had identical patterns of polymorphisms within families and sub-families, respectively. Variability in polymorphisms was observed among the isolates 656_Beijing_Bunda and 588_CAS1_Kili_Bunda which differed in patterns from their corresponding clades (see also Figure 2). The Beijing family (656 from Bunda) differed from other members of the family in at least 6 loci (Table 4**)**. This strain also differed in patterns from other Beijing strain isolates within Bunda (isolates No. 724, 725) as is also reflected in the phylogenetic dendrogram (Figure 2). The CAS1_DELHI from Ngorongoro (N1367) was also different in MIRU-VNTR typing patterns from that from Serengeti (255). The MIRU-VNTR typing showed the CAS1_Kili strains from Serengeti and Ngorongoro to have matching patterns (Table 4) (Figure 2)

## 4 Discussion

### 4.1 Spoligotyping and strain profile

The main circulating *M. tuberculosis* strains in the Serengeti ecosystem appear to be the CAS, T, LAM and the EAI genotypes in that order. These four strain families accounted for 79.4% of all genotypes, while all other named families; Beijing, Haarlem, X, U, S, MANU, accounted for only 8.9% of all genotypes. A significant percentage (11.7%) of our strains could not be linked to any known spoligotype and were therefore designated as ‘Serengeti strains’.

The predominance of the four families seen in our study is comparable with the findings of similar studies done in Kilimanjaro and Dar es Salaam, but with some differences [14,15]. Members of these families, though not in the same dominance order, have also been reported in countries neighbouring Tanzania [15], such as Ethiopia [19,26], Zambia [20] and Uganda [27–29], indicating that they are widespread in this region.

A comparison of our study findings with the other two studies previously conducted in Tanzania is shown in Table 5. The major difference between our study and the previous two studies done in Tanzania is that we found 6 (2.8%) isolates belonging to the Haarlem family while the other previous studies [14,15] did not find members of this genotype. In addition, the study by Kibiki et al. [15] did not report X and S families, while that by Eldholm et al. [14] did not report any MANU strain family. Among strain families not previously reported in Tanzania also included, EAI_Somalia, LAM3 and S/convergent and X2 subfamilies (Table 2). This study also found higher proportion of T family strains than the other two previous studies. The finding of 11.7% of genotypes with no SITs in the international spoligotype database [18] is interesting, possibly reflecting micro evolutionary events in the DR region of an existing strain [6,14,15,20,30]. The new strains (named ‘Serengeti strains’ in this study) appear to be relatively prevalent in Ngorongoro and Bunda but not in Serengeti. This variant is possibly a CAS1- Kili relative. CAS1-Kili is characterised by the absence of spacers 4–7, 10 and 20–35, whereas the Serengeti strains have additionally lost spacer 36.

**Figure 3:**
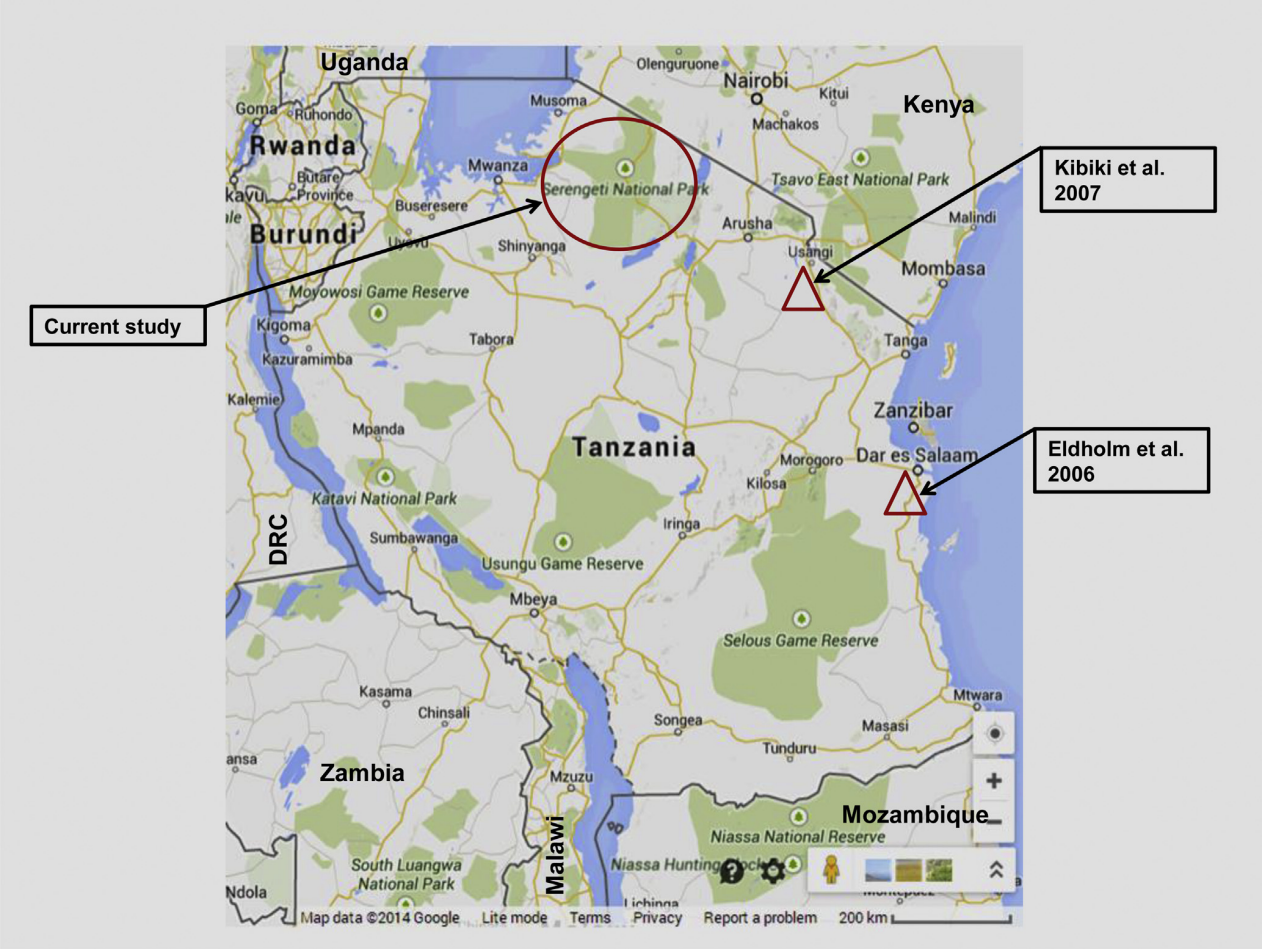
The map of Tanzania indicating where each of the three studies was conducted. The population characteristics of the three study sites differ. While the Serengeti ecosystem comprise of mostly pastoral communities, Dar es Salaam (Eldholm et al. study [14]) and Kilimanjaro (Kibiki et al. study [15]) are cities and townships with no pastoral activities at all and minimal animal-human contacts. (Map Source: www.Googlemaps.com).

Our study found some differences in the distribution of strains by districts. For example, while the T family is relatively uniformly distributed among the three districts, the CAS family predominated in Ngorongoro while EIA was highest in Bunda district. Furthermore, the lowest proportion of Beijing family was found in Ngorongoro compared to high proportions that were found in Serengeti and Bunda districts (Table 1). Other families were confined to single districts e.g. X strains were found only in Ngorongoro, S in Bunda and MANU in Ngorongoro. Comparisons of genotyping results and study sites for previous and current studies conducted in Tanzania are shown in Table 5 and Figure 3, respectively.

Further analysis (at subfamily level) of the isolates revealed the CAS1_Kili and LAM_ZWE subfamilies to be predominant in Ngorongoro, T2 and CAS1_DELHI in Serengeti and EA15 and LAM11_ZWE in Bunda (Table 2). The predominance of various strain families and subfamilies in different districts could be due to geographical isolation. CAS1_Kili for example, is believed to have emerged from the Horn of Africa, and is capable of diversifying into multiple genotypes [31]; together with other members in the CAS family they constitute the modern strains which include genetic group 1 strains belonging to the East Asian lineage (lineage 2) or to the East-African Indian lineage (lineage 3) [32]. In addition, the CAS1_Kili strain has been reported to be the dominant circulating strain in Tanzania [14,15]. It is without doubt that the CAS1_Kili has been successful in this region and as a clone it might be evolving independently acquiring genetic diversity over a long period of time thus having a high transmission level of its conserved circulating clones [33]. This could be responsible for the dominance of the CAS1_Kili family (68.4% of all CAS family) in the Ngorongoro district. We also observed variability in strain predominance with district which can be explained by differences in the characteristics of populations among the three districts. Bunda for example, is a town centre linking people in movement from various places, as such, the area is predisposed to possible introduction of potentially new strains notable in form of Beijing (varying strains of, Figure 2), S and T2-Uganda (Table 2). The changes in family strain composition in the population has been said to be attributable to increased in and out migration as well as travel across regions thus increasing chances for exposure to different strains [34]. The variability in predominance of strains in this study reflects that the Serengeti ecosystem contains a diverse group of *M. tuberculosis* strains which could have implications in devising TB control strategies. This is because the findings reflect potential for different sources of infection which could determine the type of strategy for effective control of the disease to restrain the potentially different transmission chains. This is particularly important in cases where they have different degrees of resistance against anti-tuberculosis drugs.

### 4.2 MIRU-VNTR typing and phylogenetics

In our study the dendrogram (Figure 2) from selected representative isolates that included Beijing, EAI5, CAS1_Kili, CAS1_DELHI and LAM11_ZWE showed that some of the families had identical patterns of polymorphisms located in proximity to each other in the dendrogram. The few exceptions were the 656_Beijing_Bunda and 588_CAS_Kili_Bunda that had different patterns from their corresponding clades indicative of different strains that may be new. This underlines the fact that while spoligotyping can provide rapid identification and group spoligotype patterns according to families, MIRU-VNTR typing can finely discriminate strains within and between families and subfamilies as well as establishing transmission links [20,23]. In this study, the results from MIRU-VNTR typing revealed difference in polymorphisms in at least one locus with resultant different patterns within the family as was observed in one of the Beijing families (656 from Bunda) that variably differed from other members of the family in 6 loci (similarly for other Beijing strains isolated within Bunda (isolates No. 724, 725)). The results however, showed the Beijing strains in Ngorongoro to be closely related to Beijing strains in Bunda compared to those from Serengeti. These findings demonstrate an absence of clustering which could be indicative of the importation of new Beijing strains rather than transmission. MIRU-VNTR typing also revealed the CAS1_Kili family (Table 4, Figure 2) from Serengeti and Ngorongoro to be closer to each other while differing from that from Bunda. This could mean that the circulating strains in Ngorongoro and Serengeti for CAS1_Kili have the same chain of transmission, evolution or the same recurring strain [35,36] circulating in the area. Similarly, there were variations in polymorphisms with the CAS1_DELHI isolate from Ngorongoro differing largely with that from Serengeti with polymorphisms in at least 3 loci (Table 4). Despite the few polymorphisms in those isolates that were typed, the EAI5 strains from Bunda and Serengeti differed at least in one locus, and this difference could only be revealed through MIRU-VNTR typing. Differences in patterns were also observed for the LAM11_ZWE strains from the different districts that significantly differed in at least 4 polymorphic loci.

## 5 Conclusion

This study provides for the first time, information on the prevailing human *M. tuberculosis* strains at the human–livestock–wildlife interface in the Serengeti ecosystem. Only a few successful families (CAS, T, LAM and EAI) were abundant. The other group of families that comprise Beijing, Haarlem, X, S and MANU were less frequent in this study. This study reports for the first time Haarlem, EAI_Somalia, LAM3 and S/convergent and X2 subfamilies which were not reported in previous studies in Tanzania.

## Figures and Tables

**Table 1:** Distribution of various *M. tuberculosis* families based on spoligotype pattern by district (*n* = 214).

SITs	Ngorongoro	Serengeti	Bunda	Total	%
CAS	33	11	11	55	25.7
T	14	17	21	52	24.3
LAM	18	6	14	38	17.8
EAI	6	5	14	25	11.7
Beijing	1	4	3	8	3.7
Haarlem	3	3	0	6	2.8
X	1	0	0	1	0.5
U	0	1	1	2	0.9
S	0	0	1	1	0.5
MANU	1	0	0	1	0.5
Serengeti	15	0	10	25	11.7
Total	93	46	75	214	100.0

**Table 2:** Distribution of spoligotype patterns of subfamilies in the three districts in the Serengeti ecosystem.

Sub-family	Ngorongoro	Serengeti	Bunda	Total	%
BEIJING	1	4	3	8	3.7
CAS	2	0	0	2	0.9
CAS1_DELHI	5	8	1	14	6.5
CAS1_KILI	26	3	9	38	17.8
CAS2	0	0	1	1	0.5
EAI1_SOM	1	0	0	1	0.5
EAI5	4	4	12	20	9.3
EAI5 or EAI3	1	1	2	4	1.9
H1	2	0	0	2	0.9
H3	0	3	0	3	1.4
H3-T3	1	0	0	1	0.5
LAM11_ZWE	11	6	12	29	13.6
LAM3 and S/convergent	1	0	0	1	0.5
LAM6	1	0	0	1	0.5
LAM9	5	0	2	7	3.3
MANU2	1	0	0	1	0.5
S	0	0	1	1	0.5
T1	8	3	12	23	10.7
T2	0	12	0	12	5.6
T2-T3	1	0	0	1	0.5
T2-Uganda	0	0	3	3	1.4
T3	0	0	2	2	0.9
T3_ETH	5	2	4	11	5.1
U	0	1	1	2	0.9
X2	1	0	0	1	0.5
Serengeti	15	0	10	25	11.7
Total	93	46	75	214	100.0

**Table 3:** Representative *M. tuberculosis* families as was detected by spoligotyping in this study. SITs = spoligotype international types; * = families with no (un-assigned) SITS. Black squares and white squares represent the presence and absence of specific spacer along positions 1 to 43 in the DR locus, respectively. All *M. tuberculosis* isolates were obtained from human subjects.

S/N	Family (clades)	SITs	Octal code	No. of strains	%	Spoligotype pattern	Host
1	BEIJING	1	000000000003771	08	3.7		Human
2	CAS1_KILI	21	703377400001771	38	17.8		Human
3	CAS	2269	703777740001771	02	0.9		Human
4	CAS1_DELHI	26	703777740003771	14	6.5		Human
5	CAS2	288	700377740003771	01	0.5		Human
6	EAI5	126	477777777413671	20	9.3		Human
7	EAI5 or EAI3	8	400037777413771	04	1.9		Human
8	EAI1_SOM	1801	777777777413731	01	0.5		Human
9	LAM11_ZWE	2196	777777606060771	29	13.6		Human
10	LAM6	64	777777607560771	01	0.5		Human
11	LAM9	42	777777607760771	07	3.3		Human
12	LAM3 and s/convergent	4	000000007760771	01	0.5		Human
13	H1	727	777737774020731	02	0.9		Human
14	H3	50	777777777720771	03	1.4		Human
15	H3-T3	36	777737777720771	01	0.5		Human
16	MANU2	1192	777777677763771	01	0.5		Human
17	S	34	776377777760771	01	0.5		Human
18	T1	53	777777777760771	23	10.7		Human
19	T2	135	777777777760730	12	5.6		Human
20	T2-T3	73	777737777720771	01	0.5		Human
21	T3	37	777737777760771	02	0.9		Human
22	T3_ETH	345	777000377760771	11	5.1		Human
23	T2-Uganda		437774777760730	03	1.4		Human
24	U	458	777777607740771	02	0.9		Human
25	X2	137	777776777760601	01	0.5		Human
26	Serengeti	no SITs	703377400000771	25	11.7		Human*
				214	100		

**Table 4:** Combined typing results of 24-loci MIRU-VNTR and spoligotyping analysis of 18 representative *Mycobacterium tuberculosis* isolates from the Serenegti ecosystem.

SN	Isolate	Origin	Family	MIRU-VNTR (std 24-loci)	Spoligotype patterns
1	656	Bunda	Beijing	223325163633424563544432	
2	705	Serengeti	Beijing	224325173533423463444433	
3	710	Serengeti	Beijing	224325163533423463444433	
4	700	Serengeti	Beijing	223325163533423463444433	
5	697	Serengeti	Beijing	223325163533423463344433	
6	N1254	Ngorongoro	Beijing	223325171531424553444433	
7	724	Bunda	Beijing	223335171531424553444433	
8	725	Bunda	Beijing	224325171741424553544432	
9	703	Serengeti	CAS1_Kili	229325113344524522524235	
10	481	Ngorongoro	CAS1_Kili	229325113344524322544235	
11	588	Bunda	CAS1_Kili	223525113344524322524235	
12	255	Serengeti	CAS1_DELHI	223525173423724524524455	
13	N1367	Ngorongoro	CAS1_DELHI	226525123733723523524235	
14	677	Serengeti	EAI5	245327223523642644643131	
15	B732	Bunda	EAI5	245327223523642534643131	
16	068	Ngorongoro	LAM11_ZWE	225525113324224322224253	
17	374	Bunda	LAM11_ZWE	225126141322214342244153	
18	264	Serengeti	LAM11_ZWE	225126152321312332244153	

**Table 5:** Comparison of genotyping results of our study and of two previous studies conducted in Tanzania.

Family	Eldihom et al., 2006 [18],∗	Kibiki et al., 2007 [19],∗	Current study†
CAS	52 (35.4%)	49 (37.7%)	55 (25.7%)
T	21 (14.3%)	15 (11.5%)	52 (24.3%)
LAM	33 (22.4%)	31 (23.8%)	38 (17.8%)
EAI	25 (17.0%)	13 (10.0%)	25 (11.7%)
Beijing	7 (4.8%)	7 (5.4%)	8 (3.7%)
Haarlem	0	0	6 (2.8%)
X	1 (0.7%)	0	1 (0.5%)
S	3 (2.0%)	0	1 (0.5%)
MANU	0	3 (2.3%)	1 (0.5%)
Others (Orphans)‡	5 (3.4%)	12 (9.2%)	25 (11.7%)
Total	147	130	214

∗ Only spoligotyping was done.

† Both spoligotyping and standard MIRU-VNTR were done.

‡ ‘Serengeti strains’ in the current study.
